# Evaluation of the marginal adaptation and gingival status of full-crown restorations using an intraoral camera

**DOI:** 10.1186/s12903-022-02587-3

**Published:** 2022-11-19

**Authors:** Shuting Chiu, Yeh Lee, Min Liu, Hu Chen, Hongqiang Ye, Yunsong Liu

**Affiliations:** 1 Department of Prosthodontics, Peking University School and Hospital of Stomatology & National Center of Stomatology & National Clinical Research Center for Oral Diseases & National Engineering Laboratory for Digital and Material Technology of Stomatology & Beijing Key Laboratory of Digital Stomatology & Research Center of Engineering and Technology for Computerized Dentistry Ministry of Health & NMPA Key Laboratory for Dental Materials, No. 22 Zhongguancun South Avenue, Haidian District, Beijing, 100081 People’s Republic of China; 2 Center of Digital Dentistry, Peking University School and Hospital of Stomatology & National Center of Stomatology & National Clinical Research Center for Oral Diseases & National Engineering Laboratory for Digital and Material Technology of Stomatology & Beijing Key Laboratory of Digital Stomatology & Research Center of Engineering and Technology for Computerized Dentistry Ministry of Health & NMPA Key Laboratory for Dental Materials, No. 22 Zhongguancun South Avenue, Haidian District, Beijing, 100081 People’s Republic of China

**Keywords:** Dental, Dental restorations, Digital photography, Diagnosis

## Abstract

**Objective:**

The purpose of this study was to compare the usefulness of intraoral photographs, acquired with a household intraoral camera operating in conventional, calibrated, and polarized modes, with clinical examinations for assessing the marginal adaptation and gingival status of full-crown restorations.

**Methods:**

Clinical examinations were performed by a prosthodontist who classified the marginal adaptation of full-crown restorations according to FDI World Dental Federation criteria, and a periodontal expert who classified gingival status according to the Modified Gingival Index (MGI). The margins and gingival status of the conventional, calibration, and polarization groups of full-crown restorations were independently assessed by three evaluators who obtained photographs using an intraoral camera. Cases where at least two of three assessors were in agreement were analyzed using Cohen’s kappa coefficient and the chi-square test, and the sensitivity and specificity were calculated.

**Results:**

The conventional, calibration, and polarization groups differed significantly in marginal and gingival status of full-crown restorations. In the calibration group, there was good agreement between the camera-based and oral clinical examinations in terms of the gingival status of full-crown restorations (kappa = 0.945), with 100% sensitivity and 91.67% specificity; this was also the case in the polarization group with respect to the margins of full-crown restorations (kappa = 0.917, sensitivity = 97.22%, specificity = 94.44%).

**Conclusions:**

An intraoral camera with black and white calibrated images is useful to assess the gingival status of full-crown restorations. Polarization mode can be used to assess the marginal adaptation of full-crown restorations. The camera is a feasible and valid diagnostic aid.

## Introduction

To improve long-term outcomes, crown restorations require maintenance and follow-up. Previous studies have suggested that margin misfit may cause secondary caries, gingivitis, and periodontal problems [1, 2]. However, margin quality has a greater effect on gingival health than margin position. Gingivitis is more likely to occur at the edge of a non-adherent restoration [3, 4]. The margin and irregular shape of restorations provide ideal conditions for the accumulation of food and plaque and prevent adequate oral hygiene [2, 5]. Hospital visits for clinical examinations and evaluation of full-crown restorations are necessary.

Crown restorations should be followed up within 7–10 days to evaluate function and fit, patient comfort, plaque control, and residual bonding agent in the gingival junction. A second follow-up at 6 months is recommended to check for localized defects. There are many methods to evaluate the adaptation of full-crown restorations, such as clinical direct exploration and visual examinations, x-ray radiographs, silicone replica technique, direct measurement with a microscope, etc. [6]. At present, clinical observation is mostly based on visual observation combined with probe examination and x-ray radiographs to evaluate the adaptation of full-crown restorations. Clinical examination and visual observation methods are simple, convenient, do not require other materials and equipment. However, the accuracy of these examinations are heavily dependent on clinicians’ experiences, for example, differences less than 80 μm on x-ray are difficult to observe [7].

In the past 10 years, due to oral hygiene practices, 61% of full-crown restorations had a poorly fitting margin and 80% of patients had gingivitis [8]. Due to modern lifestyles and the coronavirus 2019 (COVID-19) pandemic, many patients could not be reviewed regularly [9, 10] and teledentistry emerged as a method to provide dental care [11–13]. Therefore, innovative methods allowing self-examination by patients are urgently required for consistent and convenient follow-up. The use of digital photography in dentistry has significantly advanced in the past decade. Digital photography can be used as a clinical diagnostic tool, and there are studies on its application in diagnosing tooth decay [14–20], dental trauma [21, 22], and oral lesions [23, 24]. Signori et al. [25] compared intraoral photographs with the results of clinical evaluations of composite resin fillings and showed that digital intraoral photographs are an indirect but efficient method for assessing fillings and treatment efficacy. Meanwhile, De Almeida et al. [26] found significant differences between marginal adaptation and esthetic ratings based on clinical examination and intraoral photographs. In addition, Guo et al. [27] concluded that photographic alterations in gingival color affect clinical judgement when assessing gingival health. However, both digital and intraoral photographs exhibit color deviation and reflection, which impact the accuracy of evaluation. Kim et al. [28] developed a digital shade-matching device for dental color determination based on a support vector machine algorithm; their device uses a commercial intraoral camera, where cross-polarization is prevented by the perpendicular polarizing filter such that color distortion caused by specular reflection on the tooth surface is eliminated. However, the equipment is not commonly available or easy to use.

The objective of the present study was to compare the usefulness of portable intraoral camera photographs and intraoral clinical examinations for assessment of the margins and gingiva of full-crown restorations. The camera was operated in conventional, calibration, and polarization modes, to improve the accuracy of evaluations of the edge of full-crown restorations and gingival status. Our null hypothesis was that: (1) there are no differences in the gingival status of full-crown restorations evaluated under the conventional, calibration, and polarization modes, and (2) there are no differences in the marginal adaptation of full-crown restorations evaluated under the conventional, calibration, and polarization modes.

## Materials and methods

### Study participants

This study included 40 patients with 54 full-crown restorations seen at the Department of Prosthodontics, Peking University School and Hospital of Stomatology, China, between August and December 2021. The full-crowns were conventionally-cemented, excluding the full crown with adhesively cemented full crowns. We included patients aged ≥ 18 years with at least one premolar or molar full-crown restoration with a supragingival or flush gingival margin implanted at least 6 months prior to the study. Included patients were also without systemic disease or active periodontitis, had full behavioral autonomy, had the ability to express themselves, and exhibited good compliance. Patients were excluded if they had poor oral hygiene, acute or chronic disease in teeth adjacent to the full-crown restoration, orthodontic bracket attachments on the tooth surface, or other characteristics that may affect photography of fixed restorations.

All subjects provided written consent prior to participation. The study was performed in accordance with the Declaration of Helsinki and approved by the Biomedical Ethics Committee of Peking University Hospital of Stomatology, Beijing, China (No. PKUSSIRB-202165097).

The sample size was calculated in PASS (version 15.0, NCSS, LLC, Kaysville, UT, USA) software (α = 0.05, 80% power) according to data from the first 5 patients in this study. After calculation, a study population of at least 13 subjects was required to assess the gingival status of the full-crown restorations, and a study population of at least 29 subjects was required to assess the margins of the full-crown restorations. In the end, a total of 40 patients were recruited.

### Black and white calibration sheet

As a black and white calibration sheet, a 1.5-mm-diameter semicircle, which was half black (R:0, G:0, B:0) and half white (R:255, G:255, B:255), was printed on a piece of self-adhesive paper and calibrated. A photograph of this card, along with one of a standard black and white card, was imported into Adobe Photoshop CC 2018 (Adobe Inc., Mountain View, CA, USA) to ensure that the black and white calibration sheet was consistent with the standard color card.

### Examiners and calibration

To ensure reliability of the clinical examinations (the reference standard), the prosthodontist was trained on how to assess the marginal adaptation of full-crown restorations according to FDI World Dental Federation [29] criteria (Table 1), and the periodontist was trained in the use of the Modified Gingival Index (MGI) (Table 1) to assess the gingival status of full-crown restorations [30]. The prosthodontic and periodontal specialists who performed the clinical examinations were experts with more than 10 years of experience.

**Table 1:** FDI [29] and MGI [30] levels

	FDI		MGI
1	Harmonious outline, no gaps, no white or discolored lines	0	Absence of inflammation
2	Marginal gap (< 150 µm), white lines; small marginal fracture removable by polishing; slight ditching, slight step/flashes, or minor irregularities	1	Mild inflammation or with slight changes in color and texture, but not in all portions of marginal or papillary gingiva
3	Slight ditching, slight step/flashes, minor irregularities; non-removable gap < 250 μm; several small marginal fractures; major irregularities, ditching or step/flashes	2	Mild inflammation, such as the preceding criteria, in all portions of marginal or papillary gingiva
4	Gap > 250 μm or dentine/base exposed; severe ditching or marginal fractures; larger irregularities or steps (repair necessary)	3	Moderate, bright surface inflammation, erythema, edema and/or hypertrophy of marginal or papillary gingiva
5	Restoration (complete or partial) is loose but in situ; generalized major gaps or irregularities	4	Severe inflammation, erythema, edema and/or marginal gingival hypertrophy of the unit or spontaneous bleeding, papillary congestion, or ulceration

To ensure accurate evaluation of the full-crown restorations based on photographs obtained using an intraoral camera (Zhimei YF200B; Baden Co., Ltd., Beijing, China), three evaluators were trained. During training, the three evaluators assessed photographs similar to those evaluated in the actual study, to ensure that they understood the evaluation methods and criteria. The study photographs were assessed once the total agreement score for the three evaluators was ≥ 85% [29, 31]. The evaluators were general dentists with less than 3 years of clinical experience.

### Clinical examination

Clinical examinations were performed by trained senior clinicians (prosthodontal and periodontal experts) with more than 10 years of clinical experience. The examinations were performed on the same day, using the same dental equipment and light conditions. The instruments used for the examinations included disposable mouth mirrors and triple syringes. Prior to the examination, the teeth were cleaned with sterile gauze to remove food deposits. Teeth were examined in a wet state, but excess saliva was removed using a triple syringe if necessary [26, 32, 33]. The average time spent on the examination was almost 1 min per patient. The prosthodontist used a disposable mouth mirror to perform a visual examination to assess the margins of the full-crown restorations according to FDI [29] criteria, and the marginal adaptation of the full-crown restorations was rated as clinically acceptable (levels 1–3; 0) or unacceptable (levels 4 and 5; 1). The periodontist used a disposable mouth mirror to perform a visual examination to assess the gingival status of the full-crown restorations according to the MGI [30], and the gingival status was rated as clinically acceptable (levels 0–2; 0) or unacceptable (levels 3 and 4; 1).

### Intraoral photographs

After the clinical examination, intraoral photographs were taken under standardized conditions by a dentist trained in the use of photographic equipment [32]. The intraoral camera (Zhimei YF200B; Baden Co., Ltd.) had a 6-LED light source and provided images with a fixed resolution of 1600 × 1200. The focusing range was 10–40 mm. In order to prevent patients' cross infection, the intraoral camera was equipped with a disposable protective sheet, and the black and white calibration sheet and the polarizing filter are disposable. The patients were positioned on a dental chair, with the Frankfort plane at an angle of 45° relative to the floor. No external light source was used. The teeth were wet when photographed, but excess saliva was removed using a sterile gauze or triple syringe. The position of the full-crown restorations was determined and a black and white calibration sheet was taped over one third of the buccal side. After covering the intraoral camera with a disposable protective sheet, it was inserted into the mouth. The camera was positioned at a 45° angle relative to the buccal surfaces of the full-crown restoration, so that the full-crown restoration was located in the center of the image [25]. After conventional intraoral photographs were obtained, two linear polarizers (Edmund Industrial Optics, Barrington, IL, USA) were perpendicularly placed in front of the light source and prism to enable cross-polarization. Owing to the cross-polarization, the horizontally polarized reflected light was blocked by the perpendicularly polarizing filter, such that the color distortion caused by specular reflection on the tooth surface was eliminated [28]. Photographs taken using the intraoral camera under these conditions were categorized as polarized. After many shots, we selected photographs with a constant angular distance and evaluated for acceptability and quality; more photographs were obtained as needed.

**Fig. 1:**
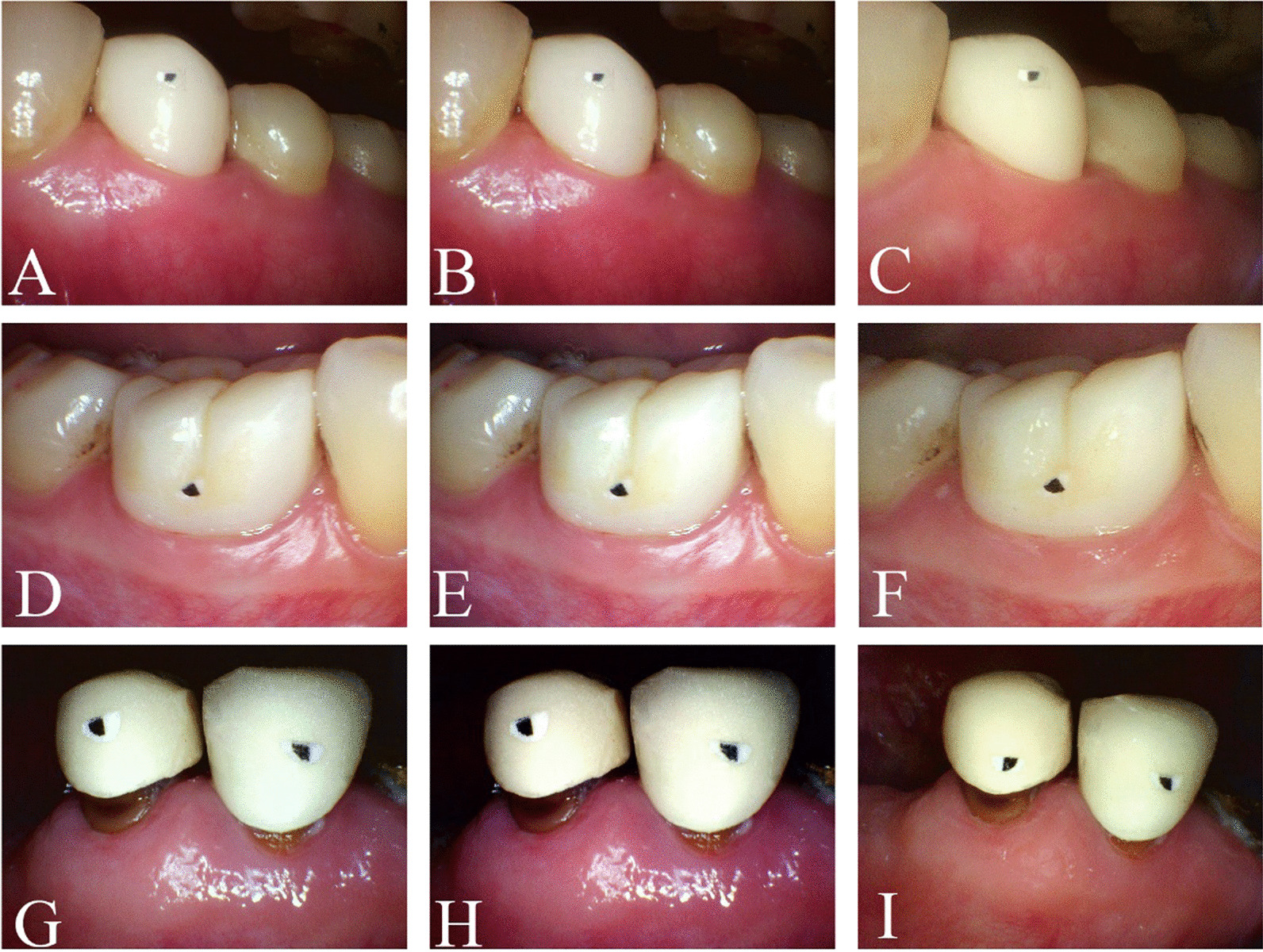
Photographs from the conventional, calibration, and polarization groups obtained using an intraoral camera. The photographs were classified into conventional (**A**/**D**/**G**), calibration (**B**/**E**/**H**), and polarization (**C**/**F**/**I**) groups (**A**/**B**/**C**, full-crown restorations with clinically acceptable margins and clinically unacceptable gingival status; **D**/**E**/**F** full-crown restorations with clinically acceptable margins and gingival status; **G**/**H**/**I** full-crown restorations with clinically unacceptable margins and gingival status)

The photographs were saved on a personal computer (Inspiron 5408; Dell Inc., Round Rock, TX, USA) as.JPG files and assigned a numerical code to protect patients’ identities [24, 30]. The photographs from the conventional intraoral photographs group were imported into Photoshop CC 2018 (Adobe) for editing and processing (of the black and white calibration). These photographs were classified as calibration photographs and stored on the personal computer as.JPG files. Sample photographs are displayed in Fig. 1.

### Photographic evaluation

Three trained dentists who did not participate in the data collection process evaluated photographs showing the full-crown restoration margins and gingival status based on the FDI [29] criteria and MGI [30] criteria. Photographs from the conventional, calibration, and polarization groups were randomly displayed on a 14-inch high-definition display. Each evaluator independently assessed the photographs for 30 s and rated the full-crown restoration margins and gingival status as clinically acceptable (0) or unacceptable (1). Cases where at least two of three assessors were in agreement were analyzed [25].

### Statistical analysis

The data were analyzed using SPSS software (version 26.0; IBM Corp., Armonk, NY, USA). The marginal adaptation and gingival status of full-crown restorations were compared among the conventional, calibration, and polarization groups. The χ^2^ test was used to compare the assessment results among the three groups; p < 0.05 was considered to indicate a statistically significant difference. The sensitivity and specificity of the camera-based assessments were calculated for each group and compared to the results of clinical examinations of the oral cavity. A confusion matrix (Table 2) summarizing the predicted and actual results was used to determine the accuracy of the model. The sensitivity and specificity were calculated as follows:

$${\text{Sensitivity:}}\quad S_{e} = \frac{a}{a + b}$$

$${\text{Specificity:}}\quad S_{P} = \frac{d}{c + d}$$

**Table 2:** Confusion matrix

Actual situation	Predicted situation	
	1	0
1	True-positive (a)	False-negative (b)
0	False-positive (c)	True-negative (d)

Cohen’s kappa statistic was used to assess the agreement between the intraoral camera-based assessments of the marginal adaptation and gingival status of full-crown restorations and the clinical examinations. Kappa values were classified as poor (≤ 0.20), fair (0.21–0.40), moderate (0.41–0.60), good (0.61–0.80), or very good (0.81–1.00) [34].

## Results

### Participant and full-crown restoration characteristics

The study included 40 patients, including 15 males and 25 females. The average age was 37.9 ± 14.31 years (22–81 years). A total of 54 full crown restorations were included, including one with 5 full crown restorations, one with 3 full crown restorations and three with 2 full crown restorations. 20 premolars and 34 molars. 30 all-ceramic crowns, 22 porcelain-fused-to-metal crowns, 2 metal crowns. Table 3 shows the detailed participant characteristics.

**Table 3:** Participant characteristics parameters

	Numeric value
Mean age	37.9 ± 14.31
Gender	
Male (n%)	15 (37.5)
Female (n%)	25 (62.5)
Tooth type	
Premolars (n%)	20 (37)
Molars (n%)	34 (63)
Full crown type	
All-ceramic crowns (n%)	30 (55.56)
Porcelain-fused-to-metal crowns (n%)	22 (40.74)
Metal crowns (n%)	2 (3.7)

### Gingival status evaluation of the conventional, calibration, and polarization groups

#### Results of the three methods for evaluating gingival status

The clinical examinations of the 54 full-crown restorations conducted by periodontal experts were considered the reference standard. In terms of successful determination of gingival status, overall, there were 43 “positive” and 11 “negative” cases. In the conventional, calibration, and polarization groups, 29, 42, and 14 cases were positive, respectively (67.44%, 97.67%, and 32.56%, respectively) (Table 4). The calibration group had the highest positivity rate, while the polarization group had the lowest positivity rate. The difference among the three groups was statistically significant (chi-square statistic = 40.522, p < 0.05). In the conventional, calibration, and polarization groups, the sensitivity and specificity values for the evaluations of gingival status of full-crown restorations were 96.67% and 41.67%, 100% and 91.67%, and 87.5% and 23.67%, respectively. The results show that the calibration group had the highest sensitivity and specificity for the evaluation of gingival state of the tooth with full-crown restoration.

**Table 4:** Results of the assessment of the gingival status of full-crown restorations in clinical and photographic evaluations

Clinical examination	Number	Conventional group		Calibration group		Polarization group	
		Positive (n/%)	Negative (n/%)	Positive (n/%)	Negative (n/%)	Positive (n/%)	Negative (n/%)
Positive	43	29 (67.44)	14 (32.56)	42 (97.67)	1 (2.33)	14 (32.56)	29 (67.44)
Negative	11	1 (9.09)	10 (90.91)	0 (0)	11 (100)	2 (18.18)	9 (81.82)

#### Comparison of the agreement between the camera-based assessments of gingival status and reference standard in the three groups

In the conventional, calibration, and polarization groups, 29, 42, and 14 results were positive, as stated above, and there was moderate (kappa = 0.405), very good (kappa = 0.945), and poor (kappa = 0.075) agreement with the reference standard, respectively (Table 5).

**Table 5:** Consistency of clinical oral and photographic evaluations of the gingival status of full-crown restorations

Group	Kappa	Level of agreement
Conventional group	0.405	Moderate
Calibration group	0.945	Very good
Polarization group	0.075	Poor

### Marginal adaptation evaluation of the conventional, calibration, and polarization groups

#### Results of the three methods for evaluating the marginal adaptation of restorations

The clinical examinations conducted by prosthodontic experts were considered the reference standard and revealed 36 “positive” and 18 “negative” cases with respect to successful determination of the marginal adaptation of the restorations. In the conventional, calibration, and polarization groups, 26 (detection rate = 72.22%), 26 (detection rate = 72.22%), and 40 (detection rate = 97.22%) cases were positive (Table 6). Thus, the positivity rate was highest in the polarization group. The difference among the three groups was statistically significant (chi-square statistic = 9.163, p < 0.05). In the conventional, calibration, and polarization groups, the sensitivity and specificity for the evaluations of marginal fitness of full-crown restorations were 92.86% and 61.54%, 92.86% and 61.54%, and 97.22% and 94.44%, respectively. Thus, the polarization group had the highest sensitivity and specificity values.

**Table 6:** Results of the assessment of the margins of full-crown restorations in clinical and photographic evaluations

Clinical examination	Number	Conventional group		Calibration group		Polarization group	
		Positive (n/%)	Negative (n/%)	Positive (n/%)	Negative (n/%)	Positive (n/%)	Negative (n/%)
Positive	36	26 (72.22)	10 (27.78)	26 (72.22)	10 (27.78)	35 (97.22)	1 (2.78)
Negative	18	2 (11.11)	16 (88.89)	2 (11.11)	16 (88.89)	1 (5.56)	17 (94.44)

#### Comparison of the extent of agreement between the camera-based and oral clinical examinations among the three methods for evaluating marginal adaptations

In the conventional, calibration, and polarization groups, 26, 26, and 34 cases were “positive”, respectively, reflecting moderate (kappa = 0.550), moderate (kappa = 0.550), and very good (kappa = 0.917) agreement with the reference standard, respectively (Table 7).

**Table 7:** Consistency of clinical oral and photographic evaluations of the margins of full-crown restorations

Group	Kappa	Level of agreement
Conventional group	0.550	Moderate
Calibration group	0.550	Moderate
Polarization group	0.917	Very good

## Discussion

The purpose of this study was to clinically analysis intraoral images acquired with a househould intraoral camera in 3 different modes. After careful assessing the marginal adaptation and gingival status of full crown restorations, the conventional, calibrated, and polarized modes demonstrated significant differences. Therefore, we reject the null hypothesis.

In the present study, we used a Zhimei intraoral camera, which is portable, inexpensive, and easy to operate. It can connect via Bluetooth and allows real-time photography and video recording. The camera was used to photograph full-crown restorations, and the level of agreement between the clinical evaluations and camera-based assessments of the margins and gingival status was moderate in the conventional group. These findings were similar to those of Signori et al. [25]. Digital photography is widely used in dental practice and allows clinicians to record the treatment process [35] and archive important information [35–37]. It also helps patients understand their clinical condition. However, conventional dental photography requires operator expertise and specialized equipment, such as oral retractors and reflectors, which may cause discomfort to patients. Compared to a conventional single-lens reflex camera, an intraoral camera can capture pictures without the need for retractors, reflectors, or other equipment. The patient can also see their oral cavity on the monitor in real time. The intraoral camera is small and convenient to use, thus reducing patient discomfort.

A black and white calibration card was used to obtain photographs with parameters close to the actual clinical evaluations. Moncada et al. [33] showed that calibration can restore the brightness, contrast, and color of images such that they match the actual clinical condition. Tobias et al. [38] proposed a dental self-timer to accurately assess gingival health through photographs. The present study evaluated the gingival status of full-crown restorations, and there was high agreement between the camera-based assessment and clinical examination in the calibration group. However, black and white calibrated images are more suited for assessing the gingival status of full-crown restorations as an indirect or complementary method.

There are many methods to evaluate the adaptation of full-crown restorations. Clinical examination and visual observation methods are simple, convenient, do not require other materials and equipment. However, the accuracy of these examinations are heavily dependent on clinicians’ experiences. To closely inspect the marginal adaptation of full-crown restorations, this study used the polarization method to prevent the reflection of light from the restoration surface, such that color parameters could be obtained more precisely [28]. Some scholars [39–41] have shown that taking color comparison photos of aesthetic restorations of the anterior teeth with oral polarization filter equipment can help to select the color of the restoration and depict details. This technique also allowed the evaluators to identify poorly fitted restorations, because differences in color between the restoration and abutment were clearly visible. There was high agreement between the camera-based and clinical evaluation results in the polarization group. Therefore, polarization mode was effective for evaluating the marginal adaptation of full-crown restorations. This study shows that the intraoral camera was effective for evaluating the marginal adaptation of full-crown restorations and associated gingival inflammation.

In the future, we hope that patients can use a standard intraoral camera to obtain photographs, thus aiding telemedical evaluation. Professionals could then make preliminary assessments and determine whether the patient needs to visit the dentist or continue with their current oral hygiene practices. Patients who do not need to visit a dentist should receive online oral hygiene guidance. Remote evaluation of pictures obtained by an intraoral camera may promote rational allocation of medical resources and improve the efficiency of medical evaluations [12, 13]. Due to the COVID-19 pandemic, many patients were unable or unwilling to attend dental facilities for regular follow-ups during the past 2 years. The use of an intraoral camera for dental evaluations could reduce the risk of infection [13, 39, 42].

Our study had a few limitations. First, the sample size was small, so we cannot definitively conclude that intraoral camera-based evaluations can replace clinical evaluations. At present, the camera should only be used as an auxiliary/supplementary evaluation method. Second, in the calibration group, a black and white calibration card had to be used on the tooth surface, while in the polarization group a linear polarizer was needed; these devices are not conducive to independent use at home. The black and white calibration film and a simpler version of the polarizer should be integrated into intraoral cameras to allow patients to obtain their own photographs. Third, at present, we are unable to evaluate the marginal adaptation and gingival status of subgingival marginal full-crown restorations. Fourth, as this study was based on two-dimensional photographs, some details may have been missed; videotaping may be preferable for remote dental evaluations.

## Conclusion

The use of a calibrated intraoral camera to assess the gingival status, and operation of the camera in polarization mode to assess the margins of full-crown restorations, are feasible and effective diagnostic aids that could facilitate the development of teledentistry.

## Data Availability

The datasets generated and/or analysed during the current study are not publicly available due to concerns about patient privacy and hospital requirements for clinical trials but are available from the corresponding author on reasonable request.

## Abbreviations

MGI: Modified Gingival Index
FDI: Foreign Direct Investment
